# Acute Perinatal Hypoxia Impairs Neurobehavioral Development and Increases Basilar Artery Contractility in Adult Male Rats

**DOI:** 10.3390/ijms27146321

**Published:** 2026-07-16

**Authors:** Anastasia A. Shvetsova, Sofia D. Kabiolskaya, Ilia A. Kabiolsky, Elena A. Sebentsova, Ekaterina K. Selivanova, Natalya G. Levitskaya, Dina K. Gaynullina

**Affiliations:** 1 Department of Human and Animal Physiology, Faculty of Biology, M.V. Lomonosov Moscow State University, 119234 Moscow, Russia; anastasiashvetsova92@gmail.com (A.A.S.); ilyakab1999@gmail.com (I.A.K.); nglevitskaya@gmail.com (N.G.L.); 2 ChemRar Research and Development Institute, 141401 Khimki, Russia; selivanova@mail.bio.msu.ru; 3 Institute of Physiology, Russian National Research Medical University, 117513 Moscow, Russia

**Keywords:** basilar artery, rho-kinase, nitric oxide, perinatal hypoxia

## Abstract

Adverse effects in early ontogenesis can have a delayed influence on the functioning of various organs. We hypothesized that acute perinatal hypoxia would worsen the neurological status and functioning of cerebral arteries in adult rats. Two-day-old male Wistar rats underwent normobaric hypoxia for 2 h (8% O_2_ content, «Hypoxia» group), while control rats from the same litters were placed in conditions with 21% O_2_ content. «Hypoxia» rats exhibited delayed maturation of motor reflexes and reduced learning ability. The levels of key serum biochemical parameters did not differ between the two groups. Contractile responses induced by the thromboxane A_2_ receptor agonist U46619 were increased in the basilar arteries of the «Hypoxia» group compared to «Control» rats. Endothelium-dependent relaxations to acetylcholine and arterial sensitivity to exogenous NO did not differ between the two groups. The NO-synthase inhibitor increased arterial contractility to a lesser extent in the «Hypoxia» group compared to the «Control» group, which was associated with decreased eNOS protein content in basilar arteries from «Hypoxia» rats. Thus, acute perinatal hypoxia impairs the neurological status of adult male rats, which may be partly associated with decreased blood supply to the brain as a result of weakened anticontractile influence of NO in basilar artery.

## 1. Introduction

An adequate supply of oxygen to organs and tissues is essential for their normal functioning. Oxygen deficiency (hypoxia) often leads to the development of pathological processes in virtually all systems of the organism. It is noteworthy that unfavorable factors in early ontogenesis can have a «programming» effect on many organ systems, and their consequences can manifest in adulthood despite the elimination of this factor [1]. One such common unfavorable factor is perinatal hypoxia, characterized by a temporary or permanent disruption of the oxygen supply to the fetus or newborn. According to clinical data, the incidence of this condition is quite high. Moreover, perinatal hypoxia is one of the leading causes of neonatal mortality [2,3,4]. Therefore, a comprehensive study of the mechanisms of delayed complications arising due to asphyxia/hypoxia in newborns is a crucial task in modern physiology and medicine.

Various organ and tissue systems are sensitive to hypoxia, but the nervous system is perhaps one of the most susceptible to the detrimental effects of hypoxia. Perinatal hypoxia has been shown to be a major cause of intrauterine damage and abnormal brain development [5]. Furthermore, a significant proportion (up to 25%) of survivors of perinatal hypoxia exhibit persistent neurophysiological impairments [6]. However, despite numerous studies, the mechanisms involved in the delayed negative effects of perinatal hypoxia remain unclear.

To reproduce cerebral damage found in children exposed to hypoxia, different animal models based on perinatal hypoxia are used. Experimental studies have shown that perinatal hypoxia disrupts sensorimotor development and cognitive functions, as well as leads to histological damage and biochemical disorders in animals [5]. Earlier, we studied acute and delayed effects of perinatal hypoxia in a model of non-invasive oxygen deprivation in rats, which recapitulates respiratory distress in preterm children. The rat pups were subjected to hypoxia on the second day of life, at a brain maturation stage that corresponds to that of humans born prematurely [7]. The rats were exposed to hypoxia for 2 h with the following gas mixture: 8% O_2_, 92% N_2_. Using this model of perinatal hypoxia, we have shown an increase in HIF1-α, glutathione peroxidase and BDNF mRNA levels as well as an increase in BDNF protein levels in the rat brain; a disturbance in the glutathione antioxidant system in blood and brain tissues; a sensorimotor impairment in early infancy; and a disruption of memory retention in adulthood [8,9]. The findings suggest that the model of neonatal hypoxia on the second day of life in rats could be used as a model of mild-to-moderate hypoxic brain damage of premature newborns.

It is important to note that the cardiovascular system is also susceptible to changes due to perinatal hypoxia. For example, we have previously demonstrated that some anticontractile mechanisms are reduced [10] and some procontractile mechanisms are increased [11] in the arteries of two-week-old rat pups exposed to hypoxia on the second day of life. These changes may potentially contribute to vasospasm and reduced blood flow.

Despite the pronounced changes observed in early ontogenesis, the delayed effects of perinatal hypoxia on vascular function have been poorly studied. Perinatal hypoxia in mice (13% O_2_ for 5 days before and 5 days after parturition) was shown to weaken the endothelium-dependent relaxation responses of adult pulmonary arteries to acetylcholine [12,13]. These changes were associated with a decrease in endothelial NO-synthase (eNOS) protein content and, accordingly, the contribution of NO to endothelium-dependent relaxation [12,13]. Prenatal hypoxia in rats (10.5% O_2_ from days 5 to 21 of pregnancy) was accompanied by an increase in the pressor response when an α1-adrenergic agonist was administered to adult animals [14]. This was accompanied by a significant increase in the contractile responses of small intestinal arteries to an α1-adrenergic receptor agonist and a decrease in large-conductance calcium-activated potassium channels (BK_Ca_) functional activity due to a decrease in the protein content of the channel’s regulatory subunit [14]. Thus, the few studies listed above demonstrate a decrease in anticontractile effects in the arteries of adult animals exposed to hypoxia in the early stages of development. Therefore, various neurological disorders resulting from perinatal hypoxia may be associated not only with the direct effects of hypoxia on nerve cells but also with impaired blood supply of the brain. However, to the best of our knowledge, the delayed effects of perinatal hypoxia on the functioning of cerebral arteries of adult organisms have never been studied before.

Therefore, we hypothesized that perinatal hypoxia would lead to neurological impairments accompanied by changes in the cerebral artery tone regulation in adult animals. To test this hypothesis, we evaluated neurobehavioral development in rats suffering from acute perinatal hypoxia and studied functional activity of the basilar artery—one of the main arteries supplying blood to the brain.

## 2. Results

### 2.1. The Influence of Acute Perinatal Hypoxia on the Physical and Sensorimotor Development of Rats

**Figure 1:**
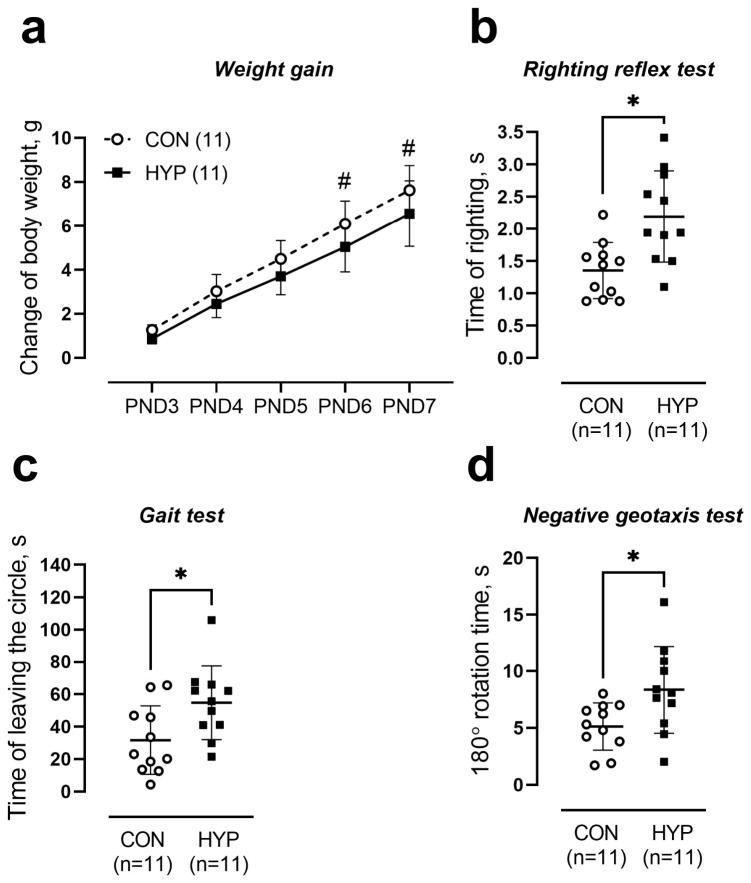
The effect of acute perinatal hypoxia on the physical and sensorimotor development of early postnatal rats. (**a**) Changes in body weight in the Control («CON») and Hypoxia («HYP») groups from PND3 to PND7. (**b**) Time to turn to the prone position (surface righting reflex). (**c**) Time to leave the circle (gait reflex). (**d**) Time to rotate from head-down position (negative geotaxis test). Numbers in parentheses indicate the number of animals in the group; rats from 7 litters were used. Data are presented as mean ± SD. # *p* < 0.05 (two-way repeated measures ANOVA followed by Sidak’s multiple comparisons test); * *p* < 0.05 (unpaired Student’s *t*-test).

No differences in initial body weight were found in the rats at the second postnatal day (PND2) before hypoxia exposure (7.9 ± 1.1 g in «Control», 7.6 ± 1.2 g in «Hypoxia», *p* > 0.05, Student’s *t*-test). After hypoxia exposure, the weight gain in the «Hypoxia» group was decreased compared to «Control» during the first postnatal week (Figure 1a, two-way ANOVA, Group factor F_1, 20_ = 6.6; *p* < 0.05). Thereafter, no effect of hypoxia on body weight was revealed (two-way ANOVA, Group factor F = 0.50; *p* > 0.45).

The maturation of the righting reflex was assessed at PND6. It showed that the surface righting latencies were significantly longer in rats of the «Hypoxia» group than in control animals (*p* < 0.05, Student’s *t*-test) (Figure 1b). A significant effect of hypoxia on the development of the gait reflex was demonstrated. At PND11, rat pups from the «Hypoxia» group required more time to leave the circle compared with control animals (*p* < 0.05, Student’s *t*-test) (Figure 1c). In addition, the rats in the «Hypoxia» group were slower to manage the negative geotaxis test compared to those in the «Control» group. The pups from the hypoxic group showed significantly longer turnaround time (*p* < 0.05) in the test than control animals (Figure 1d).

Taken together, these data suggest that acute normobaric hypoxia at PND2 in rats led to a delay in physical and sensorimotor development. Rat pups exposed to perinatal hypoxia showed impaired performance in the righting reflex, the gait reflex and the negative geotaxis test, which indicates a disorder in the formation of general motor function after hypoxia.

### 2.2. The Influence of Acute Perinatal Hypoxia on the Level of Motor and Exploratory Activity of Rats

Short-term observation of rodent behavior in an open field (OF) allows us to assess the balance between anxiety and exploratory motivation in rats [15]. On PND30, we did not observe any differences in behavior in the OF test between the rats in the two groups. There were no significant differences in parameters such as the distance traveled (41.2 ± 10.9 vs. 45.7 ± 14.9), the number of rears (11.4 ± 3.2 vs. 12.9 ± 6.7) or the number of entries to the arena center (3.5 ± 1.8 vs. 3.1 ± 1.5) in the «Control» and «Hypoxia» groups, respectively (*n* = 11 for both groups, *p* > 0.05, Student’s *t*-test). Therefore, acute normobaric hypoxia on PND2 had no influence on motor and exploratory activity in juvenile 30-day-old rats.

### 2.3. The Influence of Acute Perinatal Hypoxia on the Learning Ability of Rats

**Figure 2:**
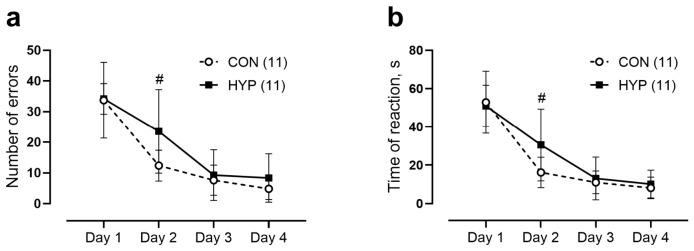
The effect of perinatal hypoxia on the acquisition of the food-reinforced complex maze task. *X*-axis—training days; *Y*-axis: (**a**)—the number of errors, (**b**)—reaction time (s) for rats of Control («CON») and Hypoxia («HYP») groups. Numbers in the parenthesis indicate the number of animals in group, the rats from 7 litters were used. Data are presented as mean ± SD. # *p* < 0.05 (two-way repeated measures ANOVA followed by Sidak’s multiple comparisons test).

The effect of perinatal hypoxia on the acquisition of the food-reinforced complex maze task was assessed from PND41 to PND44. Repeated-measures ANOVA revealed a significant main effect of factor Training day (F_3, 60_ = 106.6; *p* < 0.05 for the reaction time; F_3, 60_ = 81.38; *p* < 0.05 for the number of errors) and significant Group * Training day factor interaction (F_3, 60_ = 3.51; *p* < 0.05 for the reaction time; F_3, 60_ = 3.02; *p* < 0.05 for the number of errors). The reaction time and the number of errors in the rats of the «Hypoxia» group were higher than in «Control» animals on the second training day (*p* < 0.05, Sidak test) (Figure 2a,b). In addition, the number of correct trials on the first training day in the «Hypoxia» group (4.0; 4.0–5.0) was significantly lower than in the «Control» group (5.0; 5.0–5.0) (*p* < 0.05, Mann–Whitney test). Therefore, perinatal hypoxia leads to a delay in the acquisition of the food-reinforced complex maze task in adult rats.

### 2.4. The Influence of Acute Perinatal Hypoxia on Metabolic State of Adult Rats

To assess the delayed effect of perinatal normobaric hypoxia on the metabolic state of adult animals (11–12 week-old), the biochemical parameters characterizing, for example, liver and kidney function, were measured in the serum of two groups of rats as well as the body weight of rats was evaluated. The body weight of animals was 413 ± 58 g in the «Control» group (*n* = 11) and 377 ± 63 g in the «Hypoxia» group (*n* = 11, *p* > 0.05, Student’s *t*-test). The assessed biochemical parameters are listed in Table 1. All of the parameters had values within the normal range, while significant intergroup differences were found for alanine aminotransferase (ALT), which was slightly reduced in animals of the «Hypoxia» group compared to control rats. Thus, perinatal normobaric hypoxia used in the study had no significant influence on the general metabolic state of adult rats.

### 2.5. The Influence of Acute Perinatal Hypoxia on Contractile and Relaxatory Responses of Basilar Artery

The normalized internal diameter of the basilar arteries (d100) did not differ between the experimental groups and was 394 ± 29 μm in the «Control» group (*n* = 11) and 384 ± 25 μm in the «Hypoxia» group (*n* = 11, *p* > 0.05, Student’s *t*-test). The maximum contractile force in response to U46619 also did not differ between the groups, with values of 15 ± 5 mN in the «Control» group (*n* = 11) and 15 ± 4 mN in the «Hypoxia» group (*n* = 11; *p* > 0.05, Student’s *t*-test).

**Figure 3:**
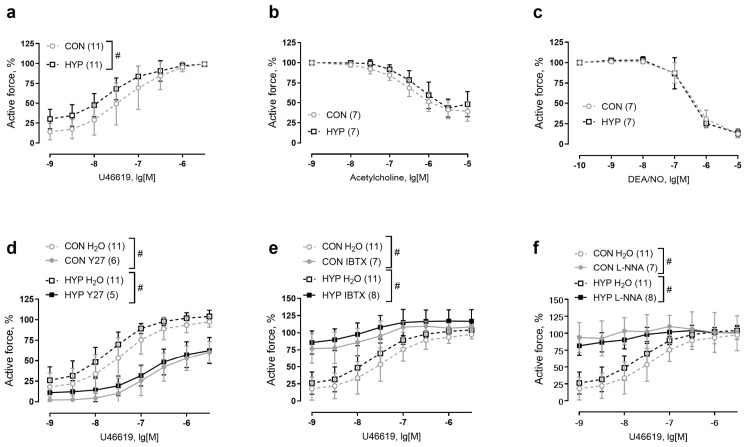
Effect of acute perinatal hypoxia on the functional activity of basilar artery. (**a**) First concentration–response relationships to U46619 of basilar arteries in rats of Control («CON») and Hypoxia («HYP») groups; rats from 7 litters were used. (**b**,**c**) Endothelium-dependent relaxation to acetylcholine (**b**) and endothelium-independent relaxation to the NO donor DEA/NO (**c**) in arteries in rats of «CON» and «HYP» groups; rats from 5 litters were used. (**d**–**f**) Second concentration–response relationships to U46619 of basilar arteries in rats of «CON» and «HYP» groups in the presence of solvent (H_2_O) or the Rho-kinase inhibitor Y27632 (Y27, (**d**), rats from 4 litters were used), the BKCa channel blocker iberiotoxin (IBTX, (**e**), rats from 5 litters were used), the NO-synthase inhibitor L-NNA ((**f**), rats from 6 litters were used). Numbers in parentheses indicate the number of animals in a group. Data are presented as mean ± SD. # *p* < 0.05 (two-way repeated measures ANOVA followed by Sidak’s multiple comparisons test). The symbol indicates statistically significant differences between groups.

At the same time, the basal tone level in the absence of vasoactive substances before the first concentration–response relationship was considerably augmented in the «Hypoxia» group and accounted for 14 ± 10% of the maximum contraction in the «Control» group (*n* = 11) and to 30 ± 12% of the maximum contraction in the «Hypoxia» group (*n* = 11, *p* < 0.05, Student’s *t*-test). In accordance with this, the contractile responses of the basilar arteries to the thromboxane A2 receptor agonist U46619 were higher in the «Hypoxia» group than in the «Control» group (Figure 3a; two-way ANOVA, Group factor: F_1, 20_ = 5.54; *p* < 0.05). Nevertheless, endothelium-dependent relaxation of the basilar artery to acetylcholine and endothelium-independent relaxation to the NO donor DEA/NO did not differ between the groups (Figure 3b: two-way ANOVA, Group factor: F_1, 12_ = 2.60; *p* > 0.05, Figure 3c: two-way ANOVA, Group factor: F_1, 12_ = 0.01; *p* > 0.05).

Next, the impacts of several important regulatory pathways (Rho-kinase, BK_Ca_ channels and eNOS) were evaluated in the arteries from two experimental groups. The Rho-kinase inhibitor Y27632 (3 μM) reduced the contractile responses of the basilar artery to U46619 in both the «Control» (two-way ANOVA with Sidak correction: t = 6.09; *p* < 0.05) and «Hypoxia» (two-way ANOVA with Sidak correction: t = 6.34; *p* < 0.05) groups (Figure 3d). In order to compare the effect of Y27632 between two groups, we calculated the area under the curve (AUC) values in the presence of the inhibitor and expressed them as a percentage of the mean AUC value in the presence of solvent in each group. In the presence of Y27632, this parameter was 40 ± 18% in control animals (*n* = 6) and 44 ± 18% in animals of the «Hypoxia» group (*n* = 5, *p* > 0.05, Student’s *t*-test).

BK_Ca_ channel blocker iberiotoxin (0.1 μM) increased contractile responses of basilar arteries to U46619 in animals of both groups (Figure 3e; two-way ANOVA with Sidak correction for «Control»: t = 5.66; *p* < 0.05 and for «Hypoxia»: t = 5.79; *p* < 0.05). In the presence of iberiotoxin, AUC increased to 155 ± 30% of the AUC in the presence of solvent in the «Control» group (*n* = 7) and to 148 ± 22% in the «Hypoxia» group (*n* = 8, *p* > 0.05, Student’s *t*-test).

NO-synthase inhibitor L-NNA (100 μM) increased contractile responses of basilar artery to U46619 in animals of the «Control» and «Hypoxia» groups (Figure 3f; two-way ANOVA with Sidak correction for «Control»: t = 6.39; *p* < 0.05 and for «Hypoxia»: t = 3.96; *p* < 0.05). In the presence of L-NNA, the elevation of AUC was significantly higher in control animals compared to hypoxia-exposed rats: AUC increased to 167 ± 35% of the AUC in the presence of solvent in the «Control» group (*n* = 7) and to 133 ± 13% in the «Hypoxia» group (*n* = 8, *p* < 0.05, Student’s *t*-test).

Thus, basilar arteries of adult rats exposed to acute normobaric hypoxia on PND2 are characterized by increased sensitivity to thromboxane A2 receptor agonist. The latter is associated with a decreased anticontractile influence of endothelial NO.

### 2.6. The Influence of Acute Perinatal Hypoxia on the Protein Content of eNOS in Basilar Artery of Adult Rats

**Figure 4:**
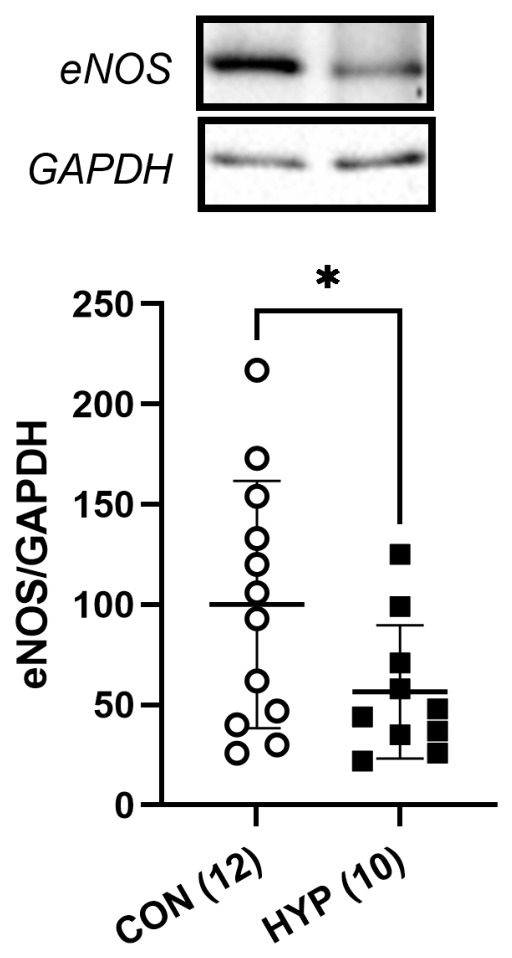
Perinatal hypoxia causes the reduction of eNOS protein content in basilar arteries of adult rats. The protein content of eNOS in basilar arteries of rats from the «Control» (CON) and «Hypoxia» (HYP) groups. The numbers in parentheses indicate the number of animals in the group; arterial samples were obtained from control and hypoxic rats from 5 litters. The protein of interest/GAPDH ratio was identified in each sample, and the average ratio in the control group was taken as 100%. Data are presented as mean ± SD. * *p* < 0.05 (unpaired Student’s *t*-test). The full-length Western blot figures are shown in Figure S1.

Finally, we checked whether the differences in the anticontractile impact of NO between basilar arteries of two experimental groups are associated with the differences in the protein content of eNOS. eNOS protein content was significantly decreased in basilar arteries of rats from the «Hypoxia» group compared to control animals (Figure 4).

## 3. Discussion

In this study, we conducted the first comprehensive assessment of the delayed effects of acute perinatal hypoxia during early ontogenesis on the neurological status of male rats and the regulation of cerebral arterial tone. We demonstrated that animals in the «Hypoxia» group exhibited delayed sensorimotor development and impaired learning ability at various stages of postnatal ontogenesis. In the vascular system, perinatal hypoxia resulted in increased basal tone and contractile responses of the basilar artery due to a decrease in the anticontractile effect of NO in adulthood. Of note, our study was performed exclusively on male rats, which is a limitation and indicates that similar studies should be performed on females in the future.

It is noteworthy that serum biochemical parameters in adult rats of the «Control» and «Hypoxia» groups were within the normal range, indicating the absence of significant metabolic violations due to hypoxia. Despite this, we demonstrated the presence of neurological deficits at various stages of ontogenesis in animals exposed to perinatal hypoxia. Our findings on the reduced learning ability and impaired sensorimotor development following perinatal hypoxia are consistent with those of other authors. In particular, an increase in reaction time in the righting reflex, gait test and negative geotaxis test has previously been demonstrated in animals exposed to perinatal hypoxia [16,17]. In addition, a number of studies indicate that sensory processing impairments (hyper- or hyposensitivity) may be both early and late consequences of hypoxic central nervous system injury in preterm infants. Studies have shown that exposure to stress (in particular, hypoxia) in early life leads to persistent microglial sensitization and neuronal death in the brain, which persists into adulthood [18]. Our findings suggest that neonatal hypoxia on the second day of life impairs the development of complex motor acts requiring limb coordination and a coordinated sensorimotor response in male rats. Our findings on learning impairments are consistent with published data, which found impaired learning with positive reinforcement in a burrow chamber in male rats that had experienced three episodes of neonatal anoxia [19]. Furthermore, hypoxia—ischemia [20], but not global asphyxia [21], was shown to impair short-term working memory in rats in the Y-maze.

Importantly, the behavioral test results correlate with changes in the regulation of basilar artery tone following perinatal hypoxia. Perinatal hypoxia resulted in increased basal tone and contractile responses of the basilar artery to a thromboxane A2 receptor agonist. In accordance with this, contractile responses of mesenteric arteries to the α1-adrenergic receptor agonist were increased in adult male rats exposed to prenatal hypoxia (10.5% O_2_ from days 5 to 21 of pregnancy) [14]. Importantly, the observed changes in vascular tone regulation appear to be region specific. Previously, using the same perinatal hypoxia model, we demonstrated no changes in the regulation of skeletal muscle arterial tone [22]. Moreover, in the coronary arteries of adult rats exposed to chronic prenatal hypoxia (10.5% O_2_ from days 5 to 21 of pregnancy), a decrease in contractile responses to serotonin was observed [23]. Despite various changes in the mechanisms regulating arterial tone in several organs and tissues, the blood pressure level in adult offspring exposed to perinatal (the same regimen used as in the present study [22]) or prenatal hypoxia [14]) did not differ from that in control animals. However, the absence of changes in blood pressure does not exclude the possibility of changes in the regulation of vascular tone in specific hemodynamic regions, in our case, the cerebral circulation.

In addition to assessing overall arterial functional activity, we investigated mechanisms associated with the procontractile effects of Rho-kinase, the anticontractile effects of BK_Ca_ channels, and endothelial NO. Previously, changes in the expression and activity of BK_Ca_ channels were shown in the pulmonary artery of adult rats subjected to prolonged neonatal hypoxia [24,25]. In contrast, a decrease in the functional activity of BK_Ca_ channels was described in a model of prolonged prenatal hypoxia in the intestinal arteries of adult rats [14]. However, our data show that acute normobaric hypoxia on the second day of life does not change the anticontractile effect of BK_Ca_ channels in the basilar artery of adult rats. The procontractile contribution of Rho-kinase to the regulation of vascular tone in light of the delayed effects of neonatal hypoxia has not previously been studied, although Rho-kinase is considered a key player in many vascular pathologies [26]. However, our results indicate that the Rho-kinase signaling pathway in the basilar artery of adult rats is not affected by acute perinatal hypoxia.

Further, we detected delayed changes in the anticontractile effect of NO in the basilar artery due to perinatal hypoxia. A decrease in the anticontractile effect of NO correlates with a decrease in the eNOS protein content in the basilar artery of animals from the «Hypoxia» group. We assume that a decrease in the anticontractile effect of NO in rats of the «Hypoxia» group is the key mechanism providing an increase in tone and contractile responses of the basilar artery. Of note, our data are consistent with the data of other authors on pulmonary circulation, according to which, when modeling prolonged perinatal hypoxia in mice, there was a decrease in eNOS protein content and the contribution of NO to endothelium-dependent relaxation in the pulmonary arteries of adult animals [12,13]. Thus, the NO pathway appears to be most susceptible to the effects of hypoxia in early ontogenesis in both cerebral and pulmonary arteries.

## 4. Materials and Methods

### 4.1. Animals

Animal studies are reported in compliance with the ARRIVE guidelines 2.0 [27,28]. Wistar rats obtained from the vivarium of the Research Institute of General Pathology and Pathophysiology were used in the present study. Sexually mature male and female rats were maintained in the laboratory animal unit of the Biological Faculty, M.V. Lomonosov Moscow State University, with a 12/12 h light/dark cycle and free access to food and water. To obtain newborn offspring, male and female rats were placed together for 4 days. In total, offspring from 8 females were used in the work. The day of birth was taken as postnatal day 0 (PND0). The next day after birth, the litters were limited to 8 pups each. Animals from each litter were kept in a separate cage with their mother until they reached 1 month of age, after which they were separated from their mothers.

On PND2, males from each litter were weighed and randomly divided into groups («Control» and «Hypoxia»). The animals of the «Hypoxia» group were then exposed to hypoxia for 2 h in a special thermostatic chamber (37 °C) supplied with a gas mixture (8% O_2_ and 92% N_2_). At this time, control animals were also removed from the nest and maintained under similar conditions of normoxia (21% O_2_). The same hypoxia conditions were used in previous studies of our group [8,9,10,11]. In order not to confuse control and hypoxic rat pups in the same litter, pups from the «Hypoxia» group were marked with a skin marker; periodically, these marks were updated. The general scheme of the experiments, indicating the timing of the tests performed and the parameters studied, is presented in Figure S2. For experiments on isolated arteries (together with blood collection for further serum biochemical measurements) rats were killed under CO_2_ anesthesia by decapitation at the age of 11–12 weeks.

During behavioral testing, the experimenter was blinded to group allocation. During myograph experiments, treatment of arterial segments with certain substances within each experimental group was randomized. Blinding of the operator was not feasible because vessel responses observed by the operator to manage the experiment permitted inferences about the treatment. When performing Western blot and measurement of serum parameters, information about samples and groups was encrypted for the operator. All data analysis was performed semi-blinded by an independent analyst.

The authors assert that all procedures contributing to this work comply with the European Convention on the protection of animals used for scientific purposes (EU Directive 2010/63/EU) and have been approved by the Biomedical Ethics Committee of M.V. Lomonosov Moscow State University (Application No 97-a-3, approved during the meeting № 149-d-3 held on 16 February 2023).

### 4.2. Behavioral Tests

To determine the rats’ physical development, their initial body weight was recorded on PND2 (before hypoxia). After hypoxic exposure, body weight was measured throughout the experiment. The weight gain was calculated as the difference between body weight at PND_N_ and body weight at PND2.

To study the influence of perinatal hypoxia on motor reflex maturation, the developmental reflexes were assessed. To test the surface righting reflex, each pup at PND6 was placed on its back in a supine position, and the time it took to roll over onto all four paws was measured. The cut-off time was 30 s. The gait reflex was assessed at PND11. The rat was placed in the center of a 13 cm diameter circle drawn on a sheet of white paper, and the time it took the rat to leave the circle with all four paws was recorded. The cut-off time was 120 s. The negative geotaxis was performed at PND12. The rat was placed on a 30 cm long inclined platform (at a 45° angle), with its head pointing downward. The turnaround time (the time required to reorient to a head-up position) was recorded. The cut-off time was 120 s.

Motor and exploratory activities were assessed using the Open Field (OF) test at PND30. The experimental setup (Open Science Research and Production Complex, Russia) consisted of a circular arena (97 cm in diameter, with walls 42 cm high). The floor was divided into 19 segments by two circles and six diameters. The experiment was conducted in silence and under red lighting. The animal was placed in the center of the arena, and within 2 min, the following parameters were recorded: total distance traveled (number of segments crossed), the number of rears, and entries to the center of the arena.

The learning ability of rats was assessed using a food-reinforced complex maze test, starting from day PND41. The complex maze is a square chamber divided into 6 corridors by five transparent partitions. Each partition had a rectangular opening that is offset relative to the openings in adjacent partitions. Before the experiment, the animals were subjected to 24 h food deprivation. On the first day of the experiment, the rats were placed in the maze for 30 min for habituation. Over the next 4 days (training days), the animal was placed in the maze 5 times in a row daily; the duration of each trial did not exceed 3 min. The following parameters were recorded daily: the number of correct trials (the number of times the animal found food reinforcement within 3 min); the number of errors (the number of any deviations from the correct movement trajectory); the reaction time. On training days, the animals were fed once a day immediately after the experiment with a limited amount of food (1/5 of the daily norm).

### 4.3. Measurement of Serum Parameters

Blood from each rat was collected right after decapitation in 1.7 mL tubes (Eppendorf). After that, the blood samples were kept for 20 min at room temperature and for 40 min at +4 C. Then they were centrifuged at 1350 g for 15 min. Liquid fraction (serum) was collected and stored at −20 °C until further analysis. Right before the measurements, the serum was defrosted, thoroughly mixed, and 500 μL of each serum sample was placed into the cone tubes. Measurement of the following parameters: alanine aminotransferase (ALT), aspartate aminotransferase (AST), alkaline phosphatase (AP), lactate dehydrogenase (LDH), gamma-glutamine transferase (GGT), total protein, albumin, globulins, Na, K, Ca, P, Cl, urea, creatinine, triglycerides and cholesterol was performed with the use of automatic biochemical analyzer VitaRay-150 (Vital Development Corporation, Saint Petersburg, Russia; JSC, China) according to the manufacturer’s instructions for corresponding biochemical kits (Vital Development Corporation, Saint Petersburg, Russia).

### 4.4. Wire Myograph Experiments

Basilar arteries were carefully cleaned from surrounding tissue in physiological salt solution (PSS) for vessel dissection ((in mM): NaCl—145; KCl—4.5; CaCl_2_—0.1; MgSO_4_—1.0; NaH_2_PO_4_—1.2; EDTA—0.025; HEPES—5.0; pH = 7.4), cut into 2 mm segments and mounted into wire myograph (620M, DMT A/S, Aarhus, Denmark) to measure isometric force. The signal was digitized at a frequency of 10 Hz using an analog-to-digital converter (E14–140, L–CARD, Moscow, Russia). Throughout the experiment, a temperature of 37 °C was maintained in the myograph chamber. Each arterial segment was stretched to 0.9d_100_ (90% of the inner diameter it would have at a transmural pressure of 100 mmHg), corresponding to maximum active force development [29]. After this, the solution in the myograph chamber was replaced with the working solution (in mM): NaCl—120; NaHCO_3_—26; KCl—4.5; CaCl_2_—1.6; MgSO_4_—1.0; NaH_2_PO_4_—1.2; D-glucose—5.5; EDTA—0.025; HEPES—5, pH = 7.4. The solution was continuously aerated with carbogen (95% O_2_ + 5% CO_2_) throughout the experiment.

At the beginning of the experiment, standard procedures for activating the preparations were performed in the following three steps by sequential application: (1) high potassium solution (60 mM of KCl); (2) U44619 (0.1 μM, thromboxane A2 receptor agonist) followed by a concentration–response relationship to acetylcholine (concentration range 1 nM–10 μM, duration of each concentration 1 min); (3) application of U46619 (1 μM). For some preparations, instead of acetylcholine, a concentration–response relationship for the NO donor DEA/NO was performed (concentration range 0.1 nM–10 μM, duration of each concentration 3 min). A washout procedure (lasting 15 min, including replacing the solution at least 3 times) was carried out after each experimental step.

The main experimental protocol consisted of two sequential concentration–response relationships for U46619 (concentration range 10 nM–3 μM, duration of action of each concentration 3 min). The first concentration–response relationship was performed 20 min after the completion of washout from the last stage of preparation activation. After washout from the first concentration–response relationship to U46619, the following substances were added to the preparations: solvent (H_2_O), Rho-kinase inhibitor Y27632 (3 µM), BK_Ca_ channel blocker iberiotoxin (0.1 µM), or NO-synthase inhibitor L-NNA (100 µM). Depending on the length of the basilar artery, the experiment was performed on three or four arterial segments, always including a control segment, to which solvent (H_2_O) was added. Twenty minutes later, a second concentration–response relationship to U46619, similar to the first one, was performed. The presence of the first concentration–response curve allowed us to confirm that arterial preparations from the same group of animals initially exhibited similar sensitivity to the agonist. This allowed the comparison of the second concentration–response relationships.

When processing the results, the value of the «passive» force, corresponding to the complete relaxation of the preparation (in the dissection solution after the procedure of optimal stretch determination), was subtracted from the force value at each concentration of the vasoactive substance. The obtained values of active force were expressed as a percentage of the maximum contractile force of the preparation, determined from the first concentration–response relationship (for U46619), or as a percentage of the precontraction level of the preparation (for acetylcholine and DEA/NO). To assess the effects of inhibitors, the values of the areas under the concentration–response curves (AUCs) were calculated using GraphPad Prism 9.5.1.

### 4.5. Measurement of Protein Abundance in Arterial Tissue by Western Blotting

Basilar arteries were isolated and quickly frozen in liquid nitrogen, where they were stored until further analysis. Arterial samples were homogenized in SDS buffer (composition: Tris-HCl (pH = 6.8), 0.0625 mM; 2.5% SDS; 10% water-free glycerin; 2.47% dithiothreitol; 0.002% bromophenol blue), supplemented with protease inhibitor cocktail (Roche, Bazel, Switzerland), centrifuged at 16,900 *g* for 5 min; supernatant was kept at −20 °C. Proteins were separated by SDS-PAGE and transferred to a nitrocellulose membrane (BioRad, Hercules, CA, USA) using the Trans-Blot Turbo transfer system (BioRad). The transfer was visualized with Ponceau S stain and the membrane was cut in two parts based on the corresponding levels of protein markers (Abcam, Cambridge, UK, cat. number 116027). All parts were blocked with 5% nonfat milk (Applichem, Darmstadt, Germany) in TBS (composition: Tris-HCl (pH = 7.6), 20 mM; NaCl, 150 mM) with 0.1% Tween 20 (TBSt). Then the lower part of the membrane was incubated overnight with antibodies against GAPDH (Abcam, cat. number 125247, mouse, 1:2000 in TBSt with 5% milk, Sigma, St. Louis, MO, USA). The upper part was incubated overnight with antibodies against eNOS (BD transduction, cat. number 610297, mouse, 1:2000 in 5% milk in TBSt). Afterwards, all membranes were incubated with antimouse secondary antibodies (Cell Signaling, Boston, MA, USA, cat. number 7076, 1:5000 in 5% milk) for 1 h and visualized with SuperSignal™ West Femto Maximum Sensitivity Substrate (Thermo Scientific, Waltham, MA, USA, cat. number 34095) using ChemiDoc (BioRad). Western blotting experiments were analyzed in ImageLab Software 6.0 (BioRad). The protein of interest/GAPDH ratio was identified in each sample, and then the average ratio in control group was taken as 100%.

### 4.6. Statistics

The minimum sample size for each experimental group was calculated using G*Power 3.1.9.7 software at a significance level of 0.05 and a type II error probability of 0.8. The results were processed statistically using GraphPad Prism 9.5.1. The normality of the data was evaluated by the Shapiro–Wilk test; homogeneity of variance was assessed by the Brown–Forsythe test. Data were analyzed for outliers using the ROUT method in GraphPad Prism 9.5.1. Depending on the type of distribution, Student’s *t*-test and 2-way repeated measures ANOVA, or the Mann–Whitney test, were applied. In the case of a significant main effect of ANOVA factors or their interaction, a post hoc analysis was carried out using the Sidak test. Differences were considered statistically significant at *p* < 0.05. Data are presented as mean ± SD or as median and interquartile range, n—number of animals per group.

## 5. Conclusions

Our results indicate that perinatal hypoxia in early ontogenesis causes neurodevelopmental impairments accompanied by changes in the regulation of basilar artery tone. A decrease in eNOS protein levels, which weakens the anticontractile effect of NO and increases basilar artery tone, may limit cerebral blood flow and serve as a risk factor for worsening the condition in cases of increased cardiovascular stress or the development of additional comorbidities.

## Figures and Tables

**Table 1:** Serum biochemistry parameters of rats from «Control» (CON) and «Hypoxia» (HYP) groups.

	CON (*n* = 7)	HYP (*n* = 7)
AST, U/L	104 (101–118)	97 (87–117)
ALT, U/L	57 (51–67)	46 (45–60) ^&^
AST/ALT	1.8 (1.6–2.4)	1.9 (1.9–2.3)
AP, U/L	173 (153–194)	168 (158–206)
LDH, U/L	583 (465–665)	524 (513–821)
GGT, U/L	3.9 (3.6–5.7)	4.0 (3.8–4.1)
Total protein, g/L	70 ± 4	67 ± 3
Albumin, g/L	43.0 ± 1.5	42.3 ± 1.4
Globulins, g/L	26.9 ± 3.0	24.9 ± 2.0
Albumin/globulins	1.61 ± 0.12	1.71 ± 0.13
Na, mM	144.1 ± 3.0	144.0 ± 0.6
K, mM	6.7 (6.4–6.8)	6.7 (6.5–7.6)
Ca, mM	2.81 (2.76–2.99)	2.82 (2.75–2.92)
P, mM	3.9 ± 0.4	4.5 ± 0.6
Cl, mM	93.2 ± 1.6	94.3 ± 1.1
Triglycerides, mM	2.2 ± 0.5	2.3 ± 0.6
Cholesterol, mM	1.91 ± 0.20	2.04 ± 0.17

AST—aspartate aminotransferase, ALT—alanine aminotransferase, AP—alkaline phosphatase, LDH—lactate dehydrogenase, GGT—gamma-glutamine transferase. Data are presented the median and the interquartile range or as the mean ± SD. Blood samples were obtained from 3 litters for «Control» group and from 3 litters for «Hypoxia» group. ^&^ *p* < 0.05 (Mann–Whitney test).

## Data Availability

The data that support the findings of this study are available from the corresponding author upon reasonable request.

## Supplementary Materials

The following supporting information can be downloaded at: https://www.mdpi.com/article/10.3390/ijms27146321/s1.

## Abbreviations

The following abbreviations are used in this manuscript:

ALT	Alanine aminotransferase
AP	Alkaline phosphatase
AUC	Area under curve
AST	Aspartate aminotransferase
eNOS	Endothelial NO-synthase
GGT	Gamma-glutamine transferase
LDH	Lactate dehydrogenase
BK_Ca_	Large-conductance calcium-activated potassium channel
OF	Open field
PND	Postnatal day
