# Supplementary material for: Acute Perinatal Hypoxia Impairs Neurobehavioral Development and Increases Basilar Artery Contractility in Adult Male Rats
Source: Int J Mol Sci. 2026 Jul 16;27(14):6321. doi: 10.3390/ijms27146321 (PMC13410226; doi:10.3390/ijms27146321)
Supplement: Supplementary file 1 [file ijms-27-06321-s001.zip › ijms-4409448-supplementary.pdf]

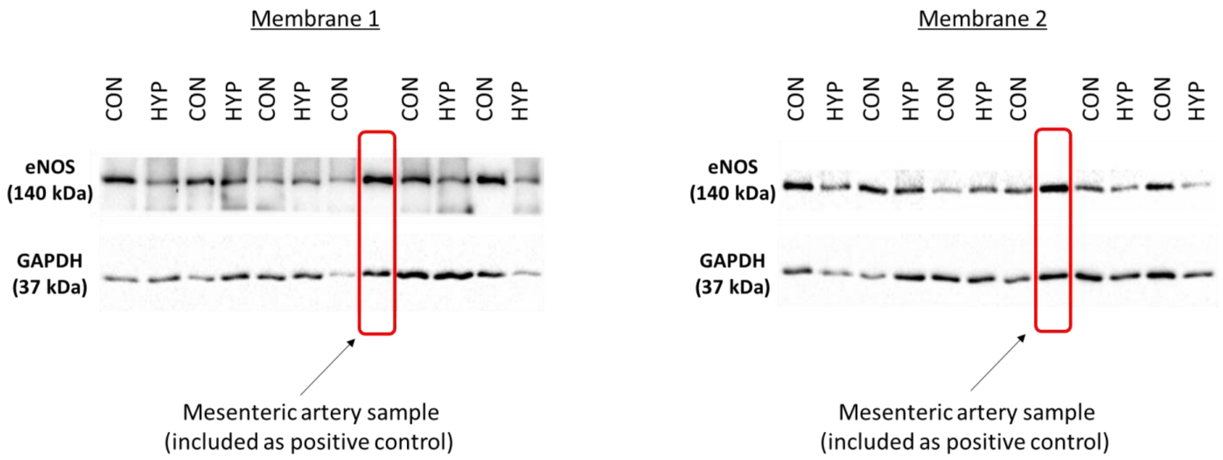

Figure S1. Original unprocessed images of Western blot membranes used in the study.

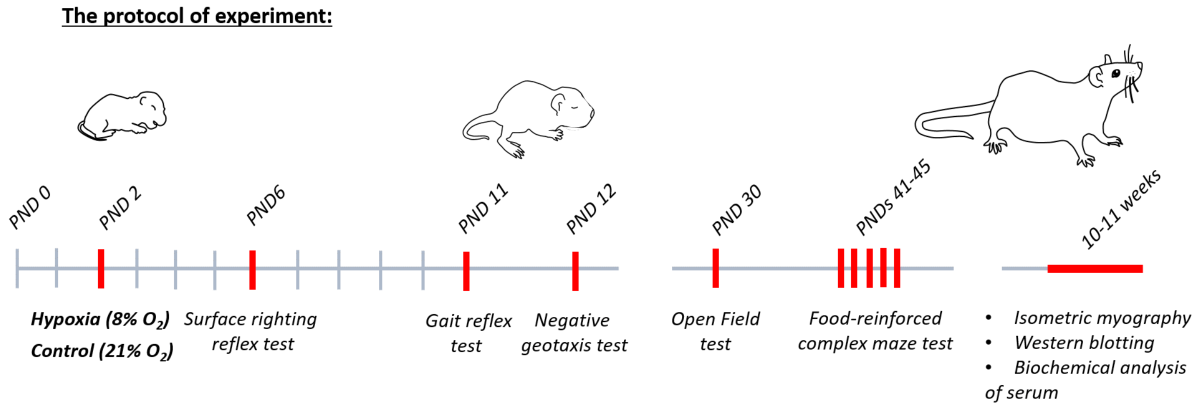

Figure S2. The general scheme of the experiment, indicating the timing of the tests performed and the parameters studied. PND – postnatal day.
